# Size compatibility and concentration dependent supramolecular host–guest interactions at interfaces

**DOI:** 10.1038/s41467-021-27659-w

**Published:** 2022-01-10

**Authors:** Jintae Park, Jinwoo Park, Jinhoon Lee, Chanoong Lim, Dong Woog Lee

**Affiliations:** School of Energy & Chemical Engineering, Ulsan National Institute of Science and Technology (UNIST), Ulsan, 44919 Republic of Korea

**Keywords:** Molecular self-assembly, Self-assembly, Organic molecules in materials science

## Abstract

The quantification of supramolecular host–guest interactions is important for finely modulating supramolecular systems. Previously, most host–guest interactions quantified using force spectroscopic techniques have been reported in force units. However, accurately evaluating the adhesion energies of host–guest pairs remains challenging. Herein, using a surface forces apparatus, we directly quantify the interaction energies between cyclodextrin (CD)-modified surfaces and ditopic adamantane (DAd) molecules in water as a function of the DAd concentration and the CD cavity size. The adhesion energy of the β-CD–DAd complex drastically increased with increasing DAd concentration and reached saturation. Moreover, the molecular adhesion energy of a single host–guest inclusion complex was evaluated to be ~9.51 *k*_B_*T*. This approach has potential for quantifying fundamental information toward furthering the understanding of supramolecular chemistry and its applications, such as molecular actuators, underwater adhesives, and biosensors, which require precise tuning of specific host–guest interactions.

## Abstract (web summary)

Quantification of supramolecular host–guest interactions is important for modulating supramolecular systems but accurately evaluating the adhesion energies of host–guest pairs remains challenging. Here, the authors directly quantify the interaction energies between cyclodextrin modified surfaces and ditopic adamantane using a surface force apparatus.

## Introduction

Biological systems are driven by various physical interactions, called noncovalent interactions, including H-bonding, van der Waals forces, electrostatic interactions, hydrophobic interactions, and metal–ligand coordination^1^. Complex combinations of these interactions lead to specific binding interactions, more commonly referred to as ligand–receptor, complementary, lock-and-key, or host–guest interactions^2,3^. The reversible nature of these interactions has inspired the development of supramolecular chemistry toward understanding biological processes such as DNA replication/transcription^4–6^ and enzyme activity^7^, which involve repeated assembly and disassembly through the highly selective recognition of specific target molecules. In particular, within supramolecular chemistry, the host–guest interaction is a crucial component for developing fundamental molecular recognition principles (e.g., the lock and key model)^8,9^. Thus, host–guest interactions have been widely investigated, from understanding molecular functions to biological applications such as hydrogels^10,11^, bioadhesives^12,13^, sensors^14,15^, and drug delivery systems^16^. Recent studies have reported that desirable properties in host–guest materials (e.g., self-healing, adhesion, and stability) can be affected by the binding affinities of host–guest inclusion complexes^17–19^.

The binding affinities of host–guest interactions have been commonly investigated using thermodynamic parameters, including the Gibbs free energy (Δ*G*) and the association constant (*K*_a_), as determined using nuclear magnetic resonance (NMR)^20^, isothermal titration calorimetry (ITC)^21^, and surface plasmon resonance (SPR)^22^. The Δ*G* and *K*_a_ values of various host–guest inclusion complexes under thermodynamic equilibrium conditions have been used as relative indicators to develop new host–guest-interaction-based materials. However, the correlation between these thermodynamic parameters and the mechanical properties of host–guest materials remains ambiguous. Thus, for furthering the understanding of host–guest interactions and expanding practical applications, measurements of the direct interaction forces and energies of host–guest inclusion complexes are essential.

The interaction forces of host–guest inclusion complexes have been investigated using single-molecule force spectroscopy (SMFS) (e.g., magnetic/optical tweezers and atomic force microscopy (AFM))^23^, which can provide the individual rupture forces of host–guest complexes at the single-molecule level^24–28^. In particular, the Vancso group studied the individual rupture forces of host–guest inclusion complexes using a β-cyclodextrin (β-CD)-modified surface as the host and a guest-immobilized AFM tip. They reported that the individual rupture force (55 pN for ferrocene–β-CD) was independent of the loading rate, the spacer chain length, and the host–guest complex concentration, which showed that the designed system was under thermodynamic equilibrium^29,30^. Furthermore, they found that the individual rupture forces (39–102 pN) measured for several types of guest molecule on the β-CD-modified surface followed the same trend as the Δ*G* values determined by ITC or SPR^31^. Similarly, Blass et al. investigated molecular kinetics and cooperative effects for host–guest complexes in terms of the rupture and friction forces measured by AFM^32–34^. In addition, they designed an energy potential model to estimate Δ*G* values from the measured forces^33^. However, the absolute Δ*G* values derived from the measured forces were significantly different from those determined by ITC or SPR owing to technical limitations such as inaccurate rupture distances, multiple interactions, and the absence of a precise and systematic model^31,33^. Thus, a different technical approach is needed for directly and accurately converting measured forces into the energies under thermodynamic equilibrium conditions.

**Fig. 1:**
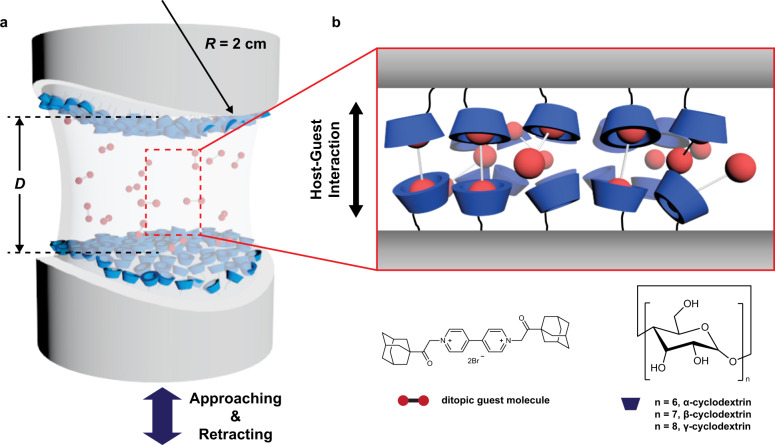
Experimental scheme. **a** Surface forces apparatus (SFA) setup for interaction force measurements. **b** Graphical illustration showing the host–guest pairs between symmetric host (CD)-modified surfaces and ditopic guest molecules (DAd).

Herein, we measured the host–guest interaction forces between β-CD and adamantane (Ad), which were selected as a representative host–guest pair with an association constant of ~10^4^ M^−1^ ^21^, using a surface forces apparatus (SFA). The SFA has been widely used to measure the absolute distances and interaction forces between macroscopic surfaces^35–37^. Compared with other SMFS techniques, the SFA has lower force resolution (~10 nN); however, in terms of accuracy and resolution for interaction energies, the SFA outperforms other force spectroscopic techniques. As the SFA utilizes a molecularly smooth surface (RMS roughness: ~0.42 Å)^38^, roughness effects can be ignored. Moreover, the SFA measures interaction forces between macroscopic surfaces with a curvature of ~2 cm, which is much larger than the working distance of the SFA (*D* < 1 μm) (Fig. 1a). As a result, the Derjaguin approximation and the Johnson–Kendall–Roberts model, which are models for converting a force between two curved surfaces to an energy per unit area, are extremely accurate^39^. Furthermore, the SFA measures the absolute distance between substrates rather than the relative displacement based on a (steric) hard wall; thus, the exact thickness of molecules at a specific applied force can be evaluated. To measure the adhesion forces between CD and Ad using the SFA, symmetric CD-modified surfaces and a ditopic adamantane (DAd) guest molecule were designed, which are expected to form CD–DAd–CD inclusion complexes in water (Fig. 1b). The formation of host–guest inclusion complexes was confirmed using 2D ROESY NMR measurements. Moreover, the surface density of grafted CD molecules was determined using a quartz crystal microbalance with dissipation monitoring (QCM-D), and the molecular interaction energy per host–guest inclusion complex was calculated.

## Results

### Synthesis of DAd as a ditopic guest molecule

**Fig. 2:**
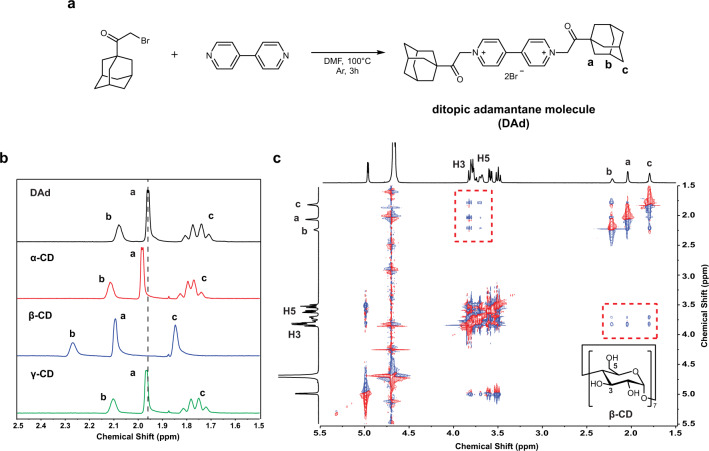
Formation of the host–guest inclusion complex in water. **a** Scheme of the synthesis of DAd. **b** ^1^H NMR spectra of the 1:2 mixtures of 1.0 mM DAd with α-, β-, and γ-CD in D_2_O. **c** 2D ROESY NMR spectrum of the 1:2 mixture of 1.0 mM DAd with β-CD in D_2_O.

To measure the host–guest interaction forces, a ditopic guest molecule was adopted to provide versatility in controlling the solution conditions between symmetric host surfaces (Fig. 1b). As a ditopic guest molecule, DAd was synthesized from 4,4′-bipyridine and 1-adamantyl bromomethyl ketone (Fig. 2a). The pyridinium salt structure of DAd ensured sufficient solubility of the hydrophobic guest molecules in water. Additionally, a short and stiff connector was required between guest moieties to achieve accurate 1:1 host–guest interactions between the opposing CD-modified surfaces. This connector prevented DAd from forming inclusion complexes with CDs within the same surface (Supplementary Fig. 1 and Supplementary Note 1). In our work, a pyridinium connector was utilized to decrease unintended intrasurface host–guest interactions^40^. The adamantly group was used as a guest moiety owing to its strong binding affinity with β-CD (*K*_a_ ~10^4^ M^−1^)^21^. The characterization of the synthesized DAd is shown in Supplementary Fig. 2.

The CD–DAd host–guest interaction was confirmed to be dependent on the size the CD cavity using NMR spectroscopy. Figure 2b shows the ^1^H NMR spectra of mixtures of DAd with α-, β-, and γ-CD. The downfield shifts observed for the methylene and methine protons of the adamantly group in the mixture of DAd with β-CD indicated the formation of a complex between the adamantly group and β-CD. The ROESY NMR spectrum of the mixture of DAd with β-CD showed correlation signals for the methylene and methine protons with H-3 and H-5 in β-CD (Fig. 2c). These results reveal that the adamantyl groups of DAd can strongly penetrate the cavity of β-CD but not those of α- and γ-CD owing to size compatibility.

### Characterization of CD surfaces

**Fig. 3:**
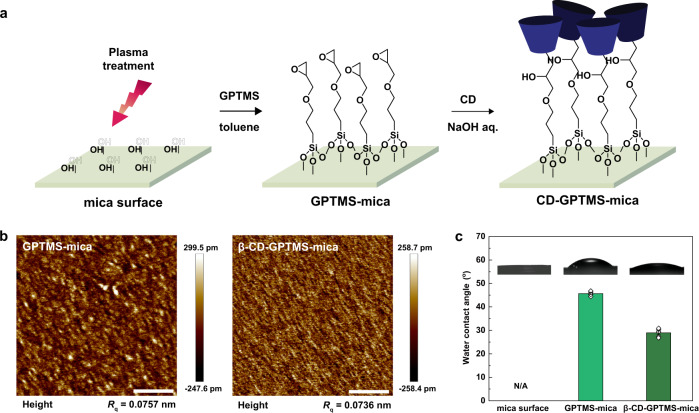
Preparation and characterization of CD-modified surfaces. **a** Schematic illustration of the procedure for preparing CD-modified mica surfaces. **b** Topographic AFM images (scale bar, 200 nm). **c** WCA measurements (error bars represent the standard error of the mean (s.e.m.), where *n* = 5). Source data are provided as a Source Data file.

To prepare the CD-modified surfaces, a molecularly smooth mica surface was functionalized with (3-glycidyloxypropyl)trimethoxysilane (GPTMS), followed by deposition of a CD layer (Fig. 3a). Topographic images of the GPTMS- and CD–GPTMS-modified surfaces were acquired using AFM tapping mode (Fig. 3b and Supplementary Fig. 3a). AFM topography analysis gave an RMS roughness of 0.074–0.092 nm for both modified surfaces, indicating the formation of homogeneous and smooth surfaces without any aggregation.

In addition, water contact angle (WCA) measurements were performed to confirm the successful modification of the surfaces with GPTMS and CD. The GPTMS-modified mica surface showed a higher WCA (45.7°) than the plasma-treated mica surface (~0°) because of the relatively hydrophobic GPTMS groups on the surface^41^. After chemically attaching CD to the surface, the WCA decreased owing to the hydrophilic hydroxyl groups of the CD molecule (Fig. 3c and Supplementary Fig. 3b). These WCA trends were comparable to those observed for GPTMS and CD monolayers in previous studies^42,43^.

### Direct interaction force measurements

We measured the force–distance profiles for two opposing CD-coated surfaces as a function of DAd concentration using the SFA. The successful grafting of CD was further confirmed by the steric wall thickness (*D*_sw_, defined as *D* at *F/R* = 40 mN m^−1^). The thickness of β-CD–GPTMS was estimated to be ~2 nm, which approximately corresponds to the sum of the GPTMS (8–10 Å) and β-CD (7.8–15.3 Å) molecular lengths^44^. Under pure distilled water (DI water) conditions, a *D*_sw_ value of ~4.4 nm was obtained, confirming that β-CD–GPTMS was well grafted to both surfaces.

**Fig. 4:**
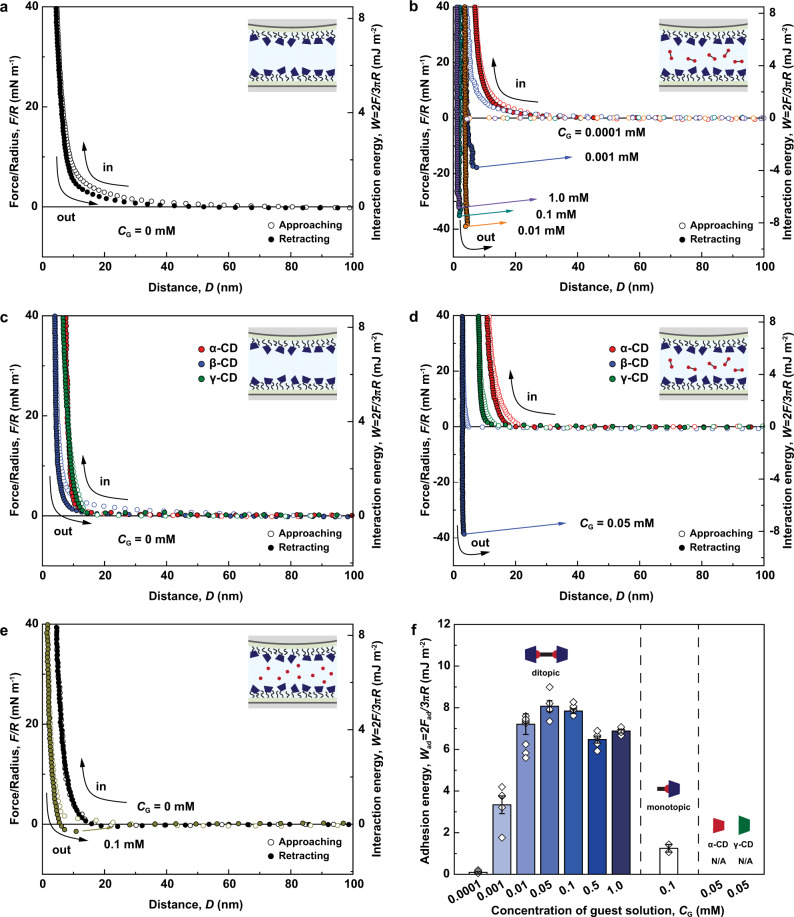
Force versus distance profiles for symmetric CD-modified surfaces and guest molecules. Symmetric β-CD-modified surfaces **a** without guest molecules and **b** with DAd at various concentrations (0.0001–1.0 mM). Symmetric CD-modified surfaces with various cavity sizes (α-, β-, and γ-CD) **c** without guest molecules and **d** with 0.05 mM DAd. **e** Symmetric β-CD-modified surface without guest molecules and with 0.1 mM MAd. **f** Overall adhesion energies (*W*_ad_) (error bars represent the s.e.m., where *n* ≥ 3). Source data are provided as a Source Data file.

Figure 4b shows the force–distance profiles for symmetric β-CD-coated surfaces as a function of DAd concentration (*C*_G_ = 0, 0.0001, 0.001, 0.01, 0.1, and 1.0 mM). Without DAd, a purely repulsive force profile was observed for the β-CD coated surfaces (Fig. 4a). The decay length of the repulsive force (*λ*^−1^) was ~15.19 nm (Supplementary Fig. 4), indicating the electrostatic repulsion between (vacant) negatively charged mica–mica surfaces upon approach. When *D* was less than 15 nm, *λ*^−1^ was ~2.14 nm, which originated from hydration repulsion^45^ and steric repulsion. In addition, no adhesion force (*F*_ad_) was observed at low *C*_G_ (<0.001 mM), indicating the existence of a critical association concentration (CAC) for DAd. Above the CAC (*C*_G_ ≥ 0.001 mM), *F*_ad_ abruptly increased with increasing *C*_G_ until reaching a maximum (*F*_ad_/*R* = 38.02 mN m^−1^, *W*_ad_ = 8.07 mJ m^−2^) at *C*_G_ = 0.05 mM, which followed the Langmuir isotherm model (*K*_a_ = 2.66 × 10^5^ M^−1^) (Supplementary Fig. 5 and Supplementary Note 2). When *C*_G_ was increased further (>0.05 mM), the adhesion plateaued or slightly decreased (Fig. 4f). This behavior indicates that all β-CD in this system was fully occupied with DAd molecules at a *C*_G_ of ~0.05 mM (Fig. 4f).

At high *C*_G_ (>0.05 mM), when the two surfaces are far apart from each other, all the β-CDs are expected to be occupied by DAd molecules with one adamantyl moiety dangling in the bulk solution (β-CD–DAd inclusion complex) (Supplementary Fig. 6a). Upon approach, this arrangement could lead to the screening of vacant β-CD sites on the opposing surface, resulting in an increase in steric repulsion between DAd molecules and a steep decrease in the bridging of DAd molecules between the two β-CD-modified surfaces (Supplementary Fig. 6b). However, the measured interactions showed only a slight decrease in adhesion (~15%) and no significant steric repulsion was observed, even when *C*_G_ was increased 20-fold to 1 mM (Fig. 4f). This discrepancy indicates that the bridging of DAd molecules between two opposing β-CD-modified surfaces (β-CD–DAd–β-CD inclusion complex) (Supplementary Fig. 6c) is more thermodynamically favorable than the complex with one dangling free end of DAd (Supplementary Fig. 6b). Nevertheless, the slight decrease in adhesion at high *C*_G_ implies that the bridging interaction was hindered by a small number of dangling adamantyl moieties.

In addition, to confirm that the CD–DAd–CD inclusion complex was in thermodynamic equilibrium, we performed adhesion force measurements as a function of contact time (*t*_c_) (Supplementary Fig. 7) and loading rate (Supplementary Fig. 8). Changes in *t*_c_ or loading velocity did not affect the bridging force of the CD–DAd–CD complex, indicating that the system reached thermodynamic equilibrium within 2 min. This result is in contrast to previous studies^46–48^ on the adhesive properties of biomacromolecules, which require long times to equilibrate and reach thermodynamic equilibrium, showing a significant increase in adhesion force and decrease in film thickness as *t*_c_ increases.

We further measured the adhesion between 1-adamantylamine (monotopic adamantane; MAd) and β-CD-modified surfaces to verify the ability of DAd molecules to bridge two β-CD-modified surfaces. For this experiment, 0.1 mM of MAd, which has the same number of adamantyl groups as 0.05 mM DAd, was injected between two β-CD-modified surfaces (Fig. 4e). As expected, only a small adhesion energy was measured (*W*_ad_ = 0.95 mJ m^−2^; less than 12% of the highest energy with DAd), suggesting that the β-CD–MAd inclusion complexes were unable to bridge two opposing surfaces.

Finally, to confirm the size dependency of the host–guest interaction between CD and Ad, force–distance profiles were obtained using surfaces modified with different types of CDs (α-, β-, and γ-CD). α-, β-, and γ-CDs have been used in various host–guest studies to evaluate cavity compatibility. At *C*_G_ = 0 mM, the measured *D*_sw_ values confirmed that the α- and γ-CD monolayers had thicknesses of ~5 nm, and the force profiles were purely repulsive, similar to that of β-CD (Fig. 4c). However, for the α- and γ-CD-modified surfaces, significant adhesion forces were not measured in the presence of DAd (*C*_G_ = 0.05 mM). Thus, the bridging interactions were maximized in the β-CD system (Fig. 4d), as expected from the CD cavity diameters (α-CD: 4.7–5.3 Å, β-CD: 6.0–6.5 Å, and γ-CD: 7.5–8.3 Å)^49^ and the size of Ad (~6.5 Å)^50^. In the case of α-CD, the DAd molecules are too large to be inserted into α-CD cavities to form bridging interactions (Supplementary Fig. 9). Instead, the DAd molecules seemed to adsorb weakly to the exterior of α-CD, inducing an increase in *D*_sw_ and a repulsive force upon compression. In contrast, although DAd can easily penetrate the large cavity of γ-CD, the cavity is too large to form stable CD–Ad inclusion complexes (Supplementary Fig. 9). Previous studies have reported that the *K*_a_ value for Ad binding to β-CD (*K*_a_ ~10^4^–10^5^ M^−1^) is more than 100 times larger than those for Ad binding to α-CD (*K*_a_ ~10^2^ M^−1^) and γ-CD (*K*_a_ ~10^2^–10^4^ M^−1^)^21,51^.

Moy et al. reported the correlation between adhesion energy (*W*_ad_) and *K*_a_ for the biotin–avidin specific interaction^52^.

1 $${W}_{{{{{{\rm{ad}}}}}}}={k}_{{{{{{\rm{B}}}}}}}T{N}_{{{{{{\rm{R}}}}}}}\,{{{{{\mathrm{ln}}}}}}\left(1+\frac{{N}_{{{{{{\rm{L}}}}}}}}{\eta {K}_{{{{{{\rm{d}}}}}}}}\right)\approx {k}_{{{{{{\rm{B}}}}}}}T{N}_{{{{{{\rm{R}}}}}}}\,{{{{{\mathrm{ln}}}}}}\left(\frac{{N}_{{{{{{\rm{L}}}}}}}}{\eta {K}_{{{{{{\rm{d}}}}}}}}\right)$$

2 $${W}_{{{{{{\rm{ad}}}}}}}\approx {k}_{{{{{{\rm{B}}}}}}}T\left(\frac{{N}_{{{{{{\rm{R}}}}}}}{N}_{{{{{{\rm{L}}}}}}}}{\eta {K}_{{{{{{\rm{d}}}}}}}}\right)$$

where *N*_R_ and *N*_L_ are the receptor and ligand densities, respectively, *η* is a proportionality constant, and *K*_d_ (or 1/*K*_a_) is the dissociation constant of the complex. They showed that in the high binding affinity regime (*K*_a_ > 10^6^ M^−1^), *W*_ad_ scales with the logarithm of *K*_a_ (Eq. (1)), whereas in the low binding affinity regime (*K*_a_ < 10^6^ M^−1^), *W*_ad_ is linearly proportional to *K*_a_ (Eq. (2)). As the *K*_a_ values for CD–Ad complexes are smaller than 10^6^ M^−1^, *K*_a_ is expected to be linearly proportional to *W*_ad_. This linear relationship is consistent with the SFA results, where the adhesion energies of α- and γ-CD with Ad are negligible compared to that of β-CD with Ad.

### Surface density measurements using QCM-D

**Fig. 5:**
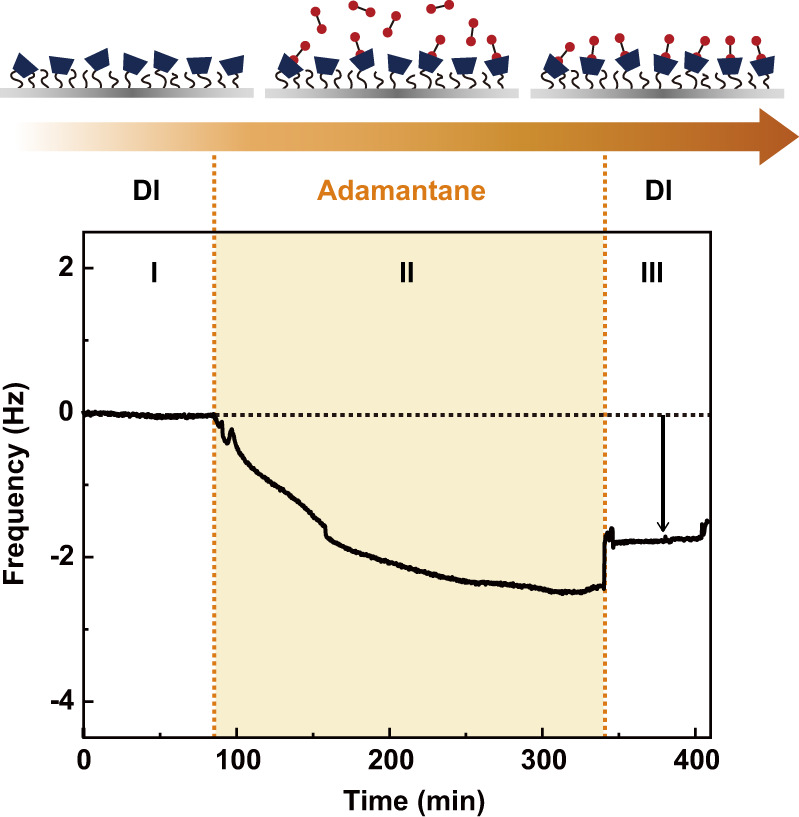
QCM-D measurement of the β-CD-modified surface. Real-time frequency shift (∆*f*) signal for a β-CD-modified QCM sensor surface in response to flowing 0.05 mM DAd aqueous solution.

We further investigated the adsorption of DAd on β-CD-modified surfaces using a QCM-D (Fig. 5). β-CD was introduced onto SiO_2_-coated quartz sensor chips through the same modification procedure as for the mica surfaces. In the QCM-D experiments, the frequency response of the β-CD-modified surface was equilibrated using DI water (I). Then, a 0.05 mM DAd aqueous solution, corresponding to the concentration that showed the highest interaction forces in the SFA experiment, was injected for 4 h (II). Finally, the β-CD-modified surface was rinsed using DI water to remove loosely bound DAd molecules (III). After injecting the DAd solution, a frequency shift of −1.30 Hz was measured, indicating the formation of the β-CD–DAd inclusion complex on the surface.

The QCM-D results showed that ∆*f* and the dissipation change (∆*D*) overlapped on all harmonics with low dispersion and that ∆*D* because almost negligible as the frequency decreased, which indicates that the surface with host–guest inclusion complexes could be considered as a rigid surface. Therefore, the Sauerbrey equation was utilized to quantify the amount of DAd adsorbed on the β-CD-modified surface, and a surface density of ~22.52 ng cm^−2^, which corresponds to 2.07 × 10^17^ molecules m^−2^, was obtained (see the Methods section for details).

### Interaction energy of a single β-CD–DAd inclusion complex

The molecular interaction energy of a single β-CD–DAd inclusion complex was calculated from the interaction energy per area (from the SFA data) and the surface density of DAd on the β-CD-modified surface (from the QCM-D data) (Table 1). The calculated single molecular energy (~9.51 *k*_B_*T*) was comparable to previously reported values determined using molecular dynamics simulations^53^ as well as ITC and SPR measurements^22^. This result demonstrates that the measured interaction force could be converted into the single molecular interaction energy without complicated procedures, thus providing a reliable *W*_ad_ value, comparable to those determined by other analytical measurements.

**Table 1:** Analysis of the interaction of DAd with β-CD-modified surfaces and the corresponding single molecular interaction energy at *C*_G_ = 0.05 mM.

Normalized force (mN m^−1^)	Adhesion energy (mJ m^−2^)	Frequency shift (Hz)	Surface density (ng cm^−2^)	Single molecular interaction energy (10^−21^ J)
38.02 ± 2.82	8.07 ± 0.60	−1.30 ± 0.06	23.11 ± 1.12	38.88 ± 3.45 (9.51 ± 0.84 *k*_B_*T*)

## Discussion

In summary, we quantified the host–guest interaction forces between symmetric β-CD-modified surfaces and DAd under aqueous conditions using an SFA. The interaction force increased drastically with increasing DAd concentration until complete host–guest complexation and then decreased slightly. Notably, with excess DAd, the interaction force was maintained owing to the rearrangement of unbound adamantyl moieties in DAd to reach thermodynamic equilibrium. Furthermore, the binding affinity of DAd with CDs was shown to depend on the CD cavity size (α-, β-, and γ-CD). As expected, a strong adhesion force was only measured for β-CD owing to the compatibility of the cavity size with Ad, as also confirmed by NMR and QCM-D measurements. Finally, the single molecular interaction energy was calculated from the interaction energy per area and the surface density determined using SFA and QCM-D measurements, respectively. The calculated single molecular interaction energy was comparable to previously reported thermodynamic parameters based on ITC and SPR measurements. These results demonstrate that the experimental approach used in this study provides not only insights into the binding energies and kinetics of host–guest interactions but also reliable parameters on the single-molecule scale that could be applied to thermodynamic systems. Therefore, we anticipate that this approach will provide fundamental information for furthering the understanding and applications of supramolecular chemistry.

## Methods

### Materials

1-Adamantyl bromomethyl ketone, GPTMS, and 1-adamantylamine were purchased from Sigma-Aldrich (USA). α-, β-, and γ-CD were purchased from Tokyo Chemical Industry Co., Ltd. (Japan). 4,4′-Bipyridine was purchased from Alfa Aesar (USA). *N,N*′-Dimethylformamide (DMF), toluene, and diethyl ether were purchased from Samchun Pure Chemical (South Korea). Sodium hydroxide (NaOH) and methanol were purchased from Daejung Chemicals & Metals Co. Ltd. (South Korea). All reagents were of analytical grade and were used as received without further purification.

### Characterization

NMR spectra (^1^H NMR, ^13^C NMR and 2D ROESY) were recorded on a 400 MHz FT-NMR spectrometer (AVANCE III HD, Bruker, USA) using DMSO-*d*_6_ or D_2_O as the solvent. The chemical shifts were referenced to the deuterated solvent. Fourier transform infrared (FTIR) spectra were recorded on a FTIR spectrometer (670-IR, Varian, USA) using an attenuated total reflection (ATR) mode. High resolution mass spectra (HRMS) analyses were performed using a direct analysis in real time (DART) method (AccuToF 4 G+ DART, JEOL, USA). The C, H, O, and N content of organic compound was determined using an elemental analyzer (Flash 2000, Thermo Scientific, USA). The surface topography was analyzed using AFM (Multimode V AFM, Veeco, USA). WCA measurements were performed using a goniometer (DSA100, KRÜSS, Germany). Droplets of ~5 µL were placed on the modified surfaces, and the WCA value was obtained as an average of at least five measurements.

### Synthesis of DAd

1-Adamantyl bromomethyl ketone (1.0 g, 3.89 mmol) and 4,4′-bipyridine (0.270 g, 1.73 mmol) were dissolved in DMF (10 mL) and allowed to react at 100 °C for 3 h under an argon atmosphere. After cooling the reaction mixture to room temperature, the yellow precipitate was collected by centrifugation at 300 *g* for 3 min. The crude product was washed with diethyl ether and recrystallized from methanol for 12 h. Subsequent drying under vacuum at 40 °C for 12 h gave the final product in 66% yield. ^1^H NMR (400 MHz, DMSO-*d*_6_): δ 9.15 (d, 4H), 8.84 (d, 4H), 6.07 (s, 4H), 2.09 (s, 6H), 1.95 (s, 12H), 1.74 (q, 12H); ^13^C NMR (100 MHz, DMSO-*d*_6_): δ 205.5, 149.1, 146.9, 126.4, 65.1, 45.3, 37.2, 35.9, 27.2; IR (ATR): 3047 cm^−1^, 2913 cm^−1^, 2854 cm^−1^, 1712 cm^−1^, 1637 cm^−1^, 1199 cm^−1^; HRMS (m/z, H_2_O, DART^−^): [C_34_H_42_N_2_O_2_Br_2_-H]^−^ calcd. for C_34_H_41_N_2_O_2_Br_2_, 669.1520; found, 669.1523; Elemental analysis (calcd., found for C_34_H_42_N_2_O_2_Br_2_ ∙ 2(H_2_O): C (57.80, 58.37), H (6.56, 6.56), N (3.96, 4.15), O (9.06, 8.96). The characterizations of the DAd were shown in Supplementary Fig. 2.

### Preparation of CD-grafted surfaces

Muscovite mica (Grade #1, S&J Trading, USA) was used as a substrate for the CD-grafted surfaces. To prepare a mica surface, freshly cleaved back-silvered mica was glued onto a cylindrical glass disc (*R* = 2 cm) using an optical adhesive (NOA 81, Norland Products Inc., USA).

The grafting process involved (i) GPTMS functionalization of the bare mica surface and (ii) CD–GPTMS grafting. For GPTMS functionalization, the glued mica surface was activated using air plasma for 3 min at 100 W under 20 Pa and a GPTMS solution (1 vol% in toluene) was added dropwise onto the activated mica surface for 30 min. Then, the mica surface was rinsed with toluene and dried with N_2_. For GPTMS–CD grafting, the GPTMS-functionalized surface was immersed in a CD solution (0.075 mg mL^−1^ α-, β-, or γ-CD in 0.1 mM NaOH) for 10 min. Subsequently, the surface was rinsed with DI water to remove unbound residues and dried with N_2_. All reactions were performed in a humidity-controlled environment (RH = 10%, *T* = 23 °C).

### Interaction force measurements using an SFA

An SFA (SFA 2000, SurForce LLC, USA) was used to measure the interaction energies and the absolute distances between the CD-grafted surfaces^54^. The CD-grafted surfaces were arranged in the SFA chamber with a cross-cylindrical geometry, and 40 µL of guest solution was injected between the surfaces. The interaction forces between the host and guest molecules were measured as a function of the guest molecule concentration (0, 0.0001, 0.001, 0.01, 0.05, 0.1, 0.5, and 1 mM DAd in DI water). In addition, to determine the effect of the guest molecule type (monotopic or ditopic) on the interaction energy, measurements were performed with 0.1 mM MAd. For each measurement, the SFA chamber was sealed with a water-soaked dust-free wiper to minimize evaporation during the experiment, and then the system was equilibrated for 1 h.

During the force measurements, the approach and retraction of two opposing surfaces were performed using a microscale motor at a constant speed (~5 nm s^−1^, otherwise mentioned). The interaction force (*F*) was measured by deflection of a double cantilever spring (*k* = 2451.7 N m^−1^) connected to the lower surface as a function of the absolute distance (*D*) between the opposing surfaces. The mica–mica distance (*D*) was confirmed using the fringes of equal chromatic order measured by multiple-beam interferometry^55^. The normalized adhesion force (*F*_ad_/*R*), which was determined using the absolute value of the minimum *F*/*R*, *F*_ad_/*R* = abs[min(*F*/*R*)], during separation, was converted to the adhesion energy per unit area (*W*_ad_ = 2*F*_ad_/3π*R*) according to the Johnson–Kendall–Roberts model^39,56^, which is the conventional method for converting measured forces to adhesion energies in SFA experiments. All force–distance measurements were conducted at room temperature (*T* = 23 °C) and repeated more than 4 times under each condition to confirm reproducibility.

Static force-runs were performed by using extra-fine control of piezoelectric tube which supports the upper disk^54^. The constant voltage steps of 1.0 V was applied to the piezoelectric crystal to move the upper surface with regular shifts of distance (~5 nm). The surfaces were equilibrated at each distance (*D*) for ~40 s before moving to next *D*. Voltage-to-distance calibration of piezoelectric crystal was evaluated at separated distance (*D* > 400 nm), where no interaction forces were measured. All static force-runs were repeated 3 times for reproducibility.

### Surface density measurements using QCM-D

The adsorption of guest molecules on the host-modified surfaces was investigated using QCM-D (QCM-D E4, Q-Sense, Sweden). The SiO_2_-coated quartz sensor chip (QSX 303, Q-Sense, Sweden) was cleaned by UV–ozone treatment for 10 min, rinsed twice with ethanol, and dried with N_2_. Then, the surface of the sensor chip was modified with β-CD using the same surface modification procedure as for the mica surface. All experiments were performed at a constant flow rate of 50 µL min^−1^ and a constant temperature of 25 °C. The monitored shifts in ∆*f* and ∆*D* were analyzed using the QTools software (Q-Sense, Sweden). The baseline drift was subtracted from the data. The surface density (∆*m*) was calculated from the negative shift in ∆*f* using the Sauerbrey equation^57^:

3 $$\varDelta m=-\frac{\sqrt{{\rho }_{{{{{{\rm{q}}}}}}}{\mu }_{{{{{{\rm{q}}}}}}}}}{2{f}_{0}^{2}}\frac{\varDelta {f}_{{{{{{\rm{n}}}}}}}}{n}=-\frac{C\varDelta {f}_{{{{{{\rm{n}}}}}}}}{n}$$

where *ρ*_q_ is the density of quartz (2.648 g cm^−3^), µ_q_ is the shear modulus of quartz for an AT-cut crystal (29.47 GPa), *f*_0_ is the resonant frequency of the fundamental mode (5 MHz), *C* is the mass sensitivity constant (17.7 ng cm^−2^ Hz^−1^), and *n* is the overtone number^58^. In this work, the seventh overtone was adopted for this calculation.

## Supplementary information

Supplementary Information

## Data Availability

The authors declare that all data supporting the findings of this study are available within the article and its supplementary information files. Additional data related to this study can be requested from the corresponding author upon request. Source data are provided with this paper.

## Source data

Source Data
