# Supplementary material for: Size compatibility and concentration dependent supramolecular host–guest interactions at interfaces
Source: Nat Commun. 2022 Jan 10;13:112. doi: 10.1038/s41467-021-27659-w (PMC8748952; doi:10.1038/s41467-021-27659-w)
Supplement: Supplementary file 1 — Supplementary Information [file 41467_2021_27659_MOESM1_ESM.pdf]

Supplementary Information

Size compatibility and concentration dependent supramolecular host–guest interactions at interfaces

Jintae Park[1,2], Jinwoo Park[1,2], Jinhoon Lee[1], Chanoong Lim[1]* & Dong Woog Lee[1]*

[1]School of Energy & Chemical Engineering, Ulsan National Institute of Science and Technology (UNIST), Ulsan 44919, Republic of Korea

[2]These authors contributed equally: Jintae Park, Jinwoo Park

*email: c.lim@unist.ac.kr; dongwoog.lee@unist.ac.kr

Contents

Supplementary Figures 1

Supplementary Fig. 1 Scheme of the possible intra-surface CD-DAd-CD bridging 1

Supplementary Fig. 2 Characterization of ditopic adamantane molecule (DAd) 2

Supplementary Fig. 3 Characterization of α- and γ-CD-modified mica surfaces 3

Supplementary Fig. 4 Semi-log force profile between β-CD vs. β-CD 3

Supplementary Fig. 5 Formation of the host–guest complex at the β-CD-modified surface 4

Supplementary Fig. 6 Interaction mechanism between β-CD-modified surfaces and DAd 5

Supplementary Fig. 7 Effect of contact time (t_c) on the interaction forces 6

Supplementary Fig. 8 Effect of loading rate on the interaction forces 7

Supplementary Fig. 9 Host–guest interaction between α-, β-, and γ-CD-modified surfaces and DAd 8

Supplementarty Notes 9

Supplementary Note 1 Possibility of intra-surface CD-DAd-CD bridging 9

Supplementary Note 2 Equilibrium of host-guest inclusion complex 10

Supplementary References 12

Supplementary Figures

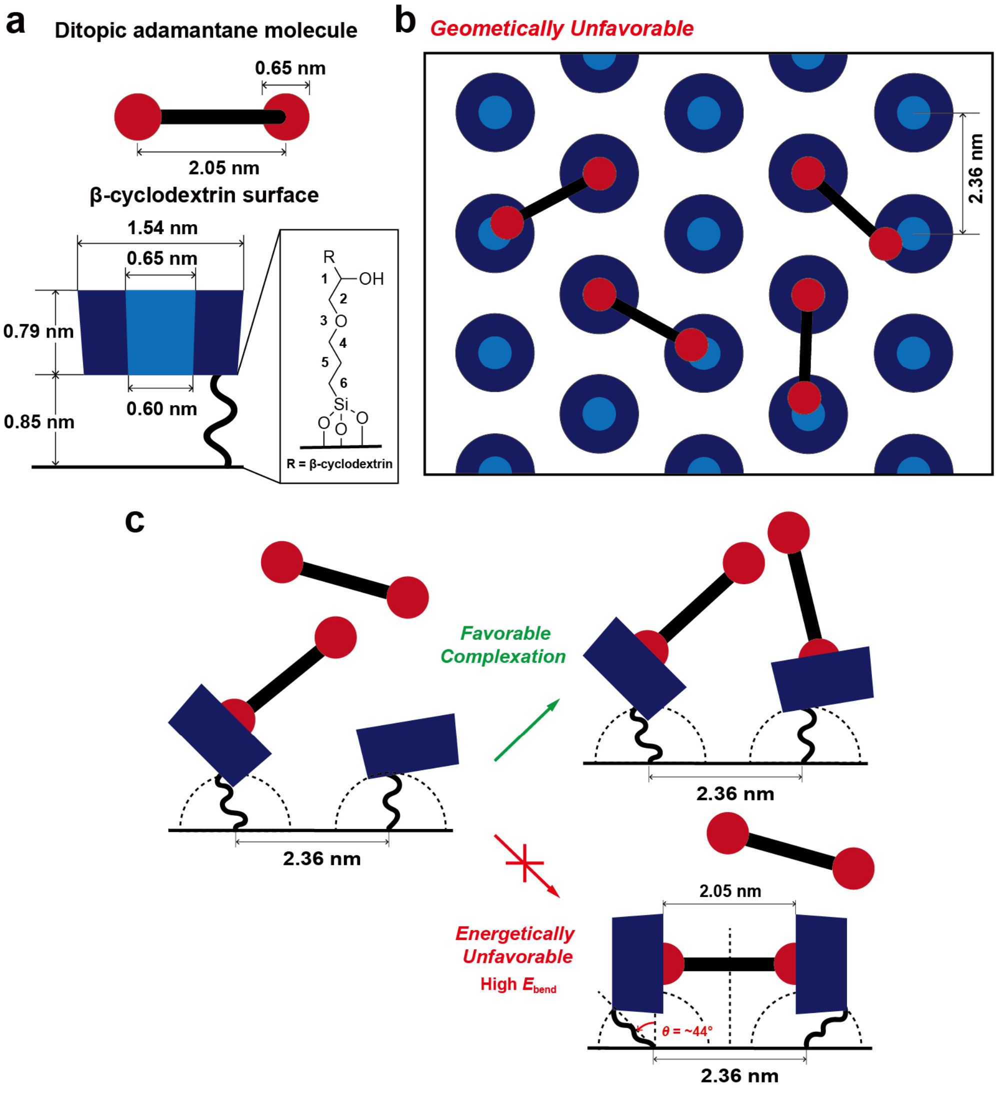

Supplementary Fig. 1 Scheme of the possible intra-surface CD-DAd-CD bridging. a Chemical structure of GPTMS linker and dimensions of ditopic adamantane molecule and β-cyclodextrin surface.[1,2] **b** Top view and **c** side view of β-cyclodextrin surface with ditopic guest molecules. The intra-surface bridging is suppressed due to high bending strain at GPTMS linker.

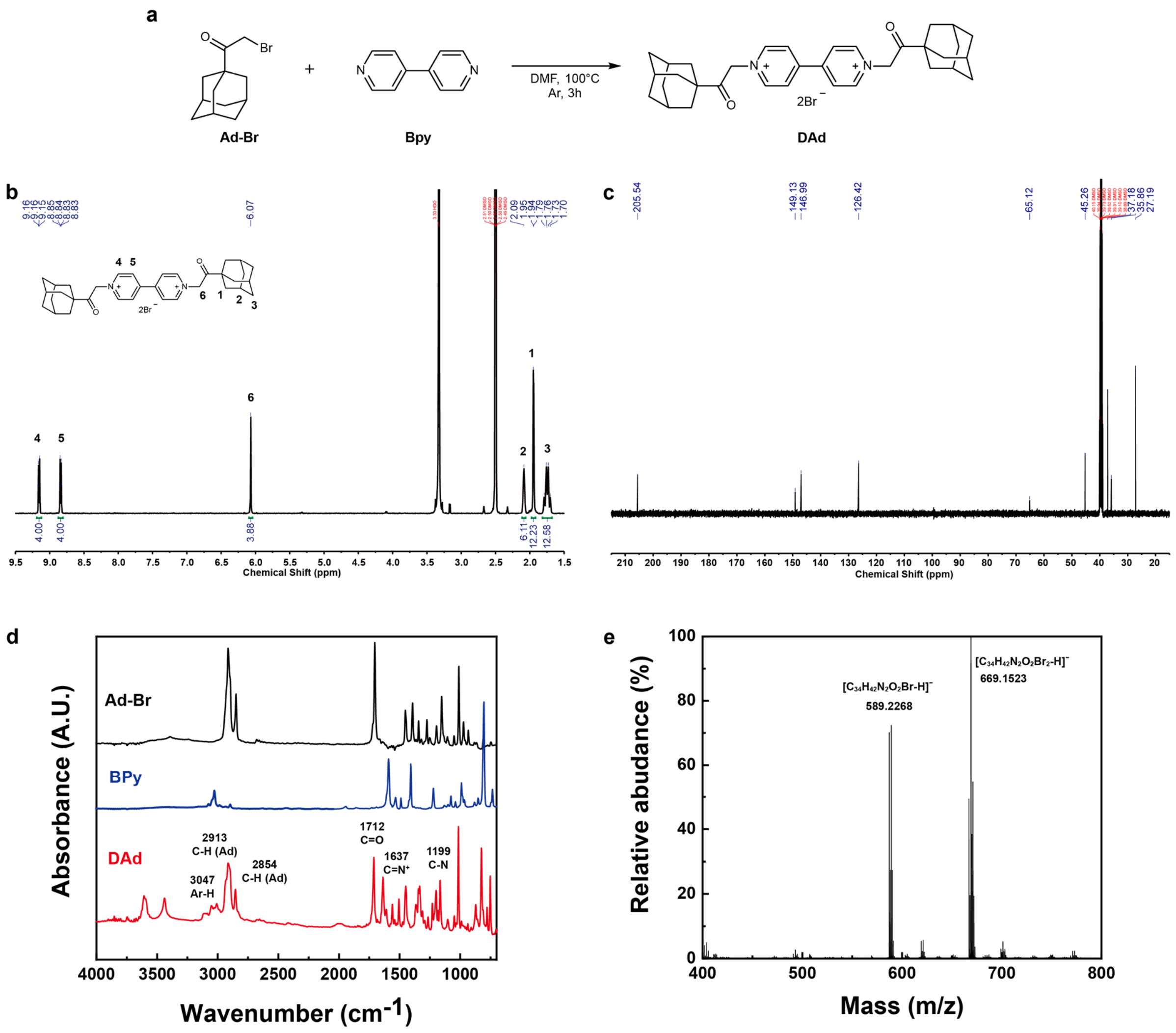

Supplementary Fig. 2 Characterization of ditopic adamantane molecule (DAd). a Scheme of the synthesis of DAd. **b** ^{1}H NMR spectrum (400 MHz, DMSO-d_6) of DAd. **c** ^{13}C NMR spectrum (100 MHz, DMSO-d_6) of DAd. **d** ATR-FTIR spectra of Ad-Br, Bpy, and DAd. **e** DART-HRMS spectrum of DAd.

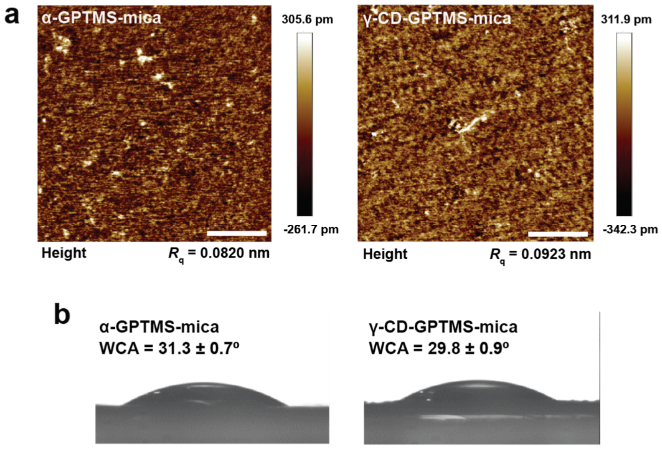

Supplementary Fig. 3 Characterization of α- and γ-CD-modified mica surfaces. a Topographic AFM images (scale bar, 200 nm). **b** Water contact angle measurements (mean ± standard error of the mean (s.e.m.), where n = 5).

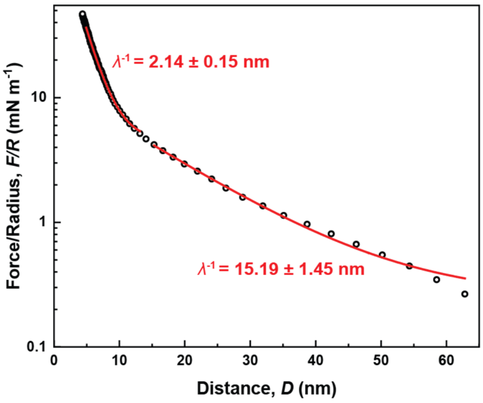

Supplementary Fig. 4 Semi-log force profile between β-CD *vs.* β-CD. Repulsive forces between symmetric β-CD-modified surfaces in pure DI water with a decay length of ~15.19 nm (at D > 15 nm) and ~2.14 nm (at D < 15 nm).

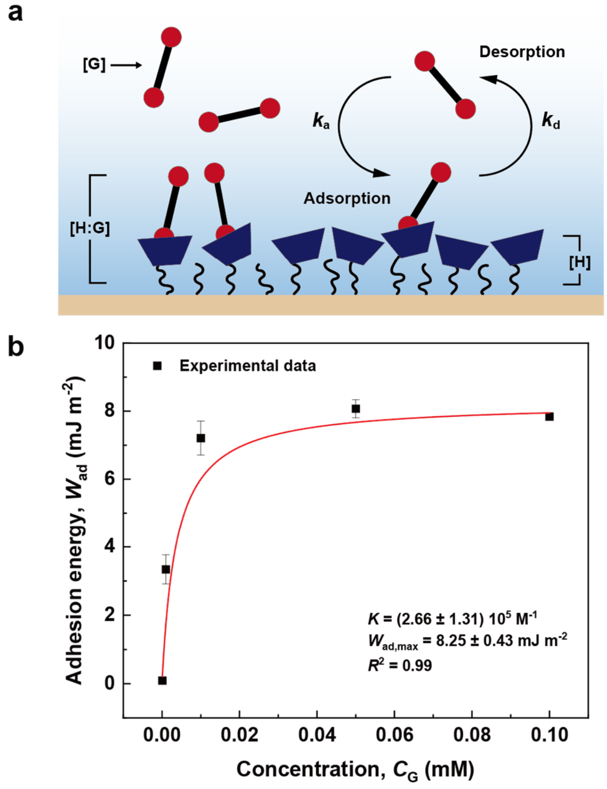

Supplementary Fig. 5 Formation of the host–guest complex at the β-CD-modified surface. a Scheme of equilibrium process at the host surface in the aqueous guest molecule solution. **b** Adhesion energy as a function of the concentration of guest molecule. The red line represents the curve fitted by Supplementary Note 2 and Supplementary Equation (1). The error bars represent the s.e.m., where $n \geq 4$.

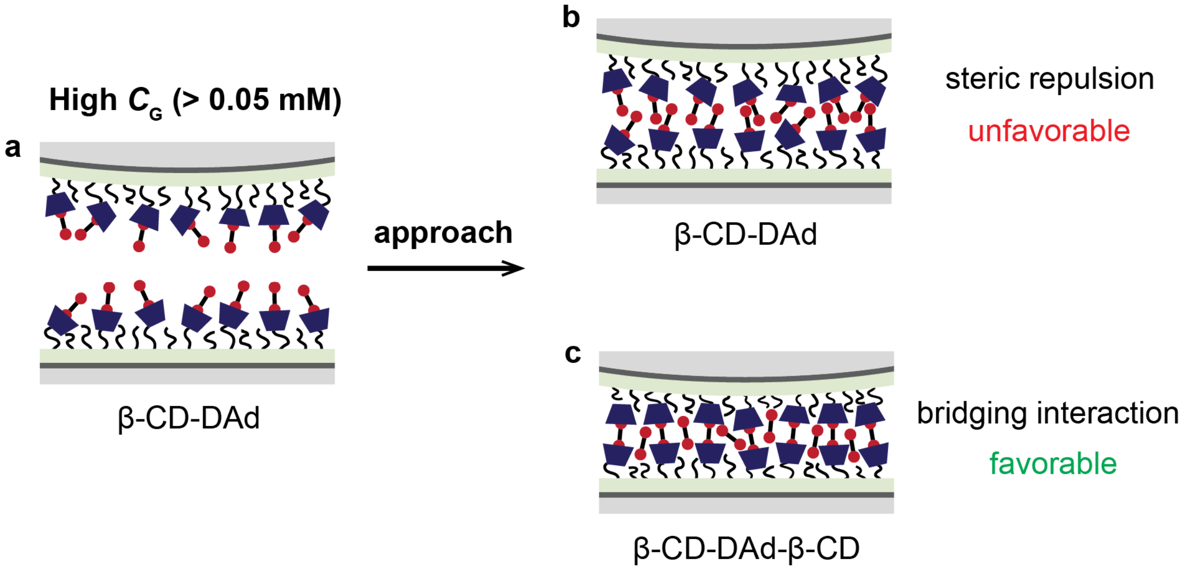

Supplementary Fig. 6 Interaction mechanism between β-CD-modified surfaces and DAd. a β-CD–DAd inclusion complex on the opposing surfaces. **b** Unfavorable interaction model by steric repulsion. **c** Ideal bridging interaction model.

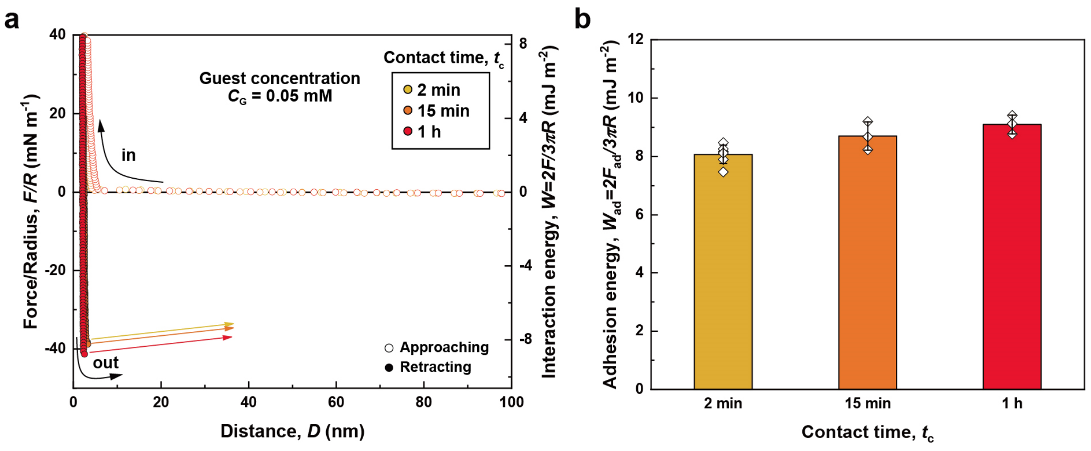

Supplementary Fig. 7 Effect of contact time (t_c) on the interaction forces. a Force-distance profile of symmetric β-CD-modified surfaces with 0.05 mM of DAd at different t_c (2 min, 15 min, and 1 h). **b** Adhesion energy as a function of t_c (the error bars represent the s.e.m., where $n \geq 3$). Source data are provided as a Source Data file.

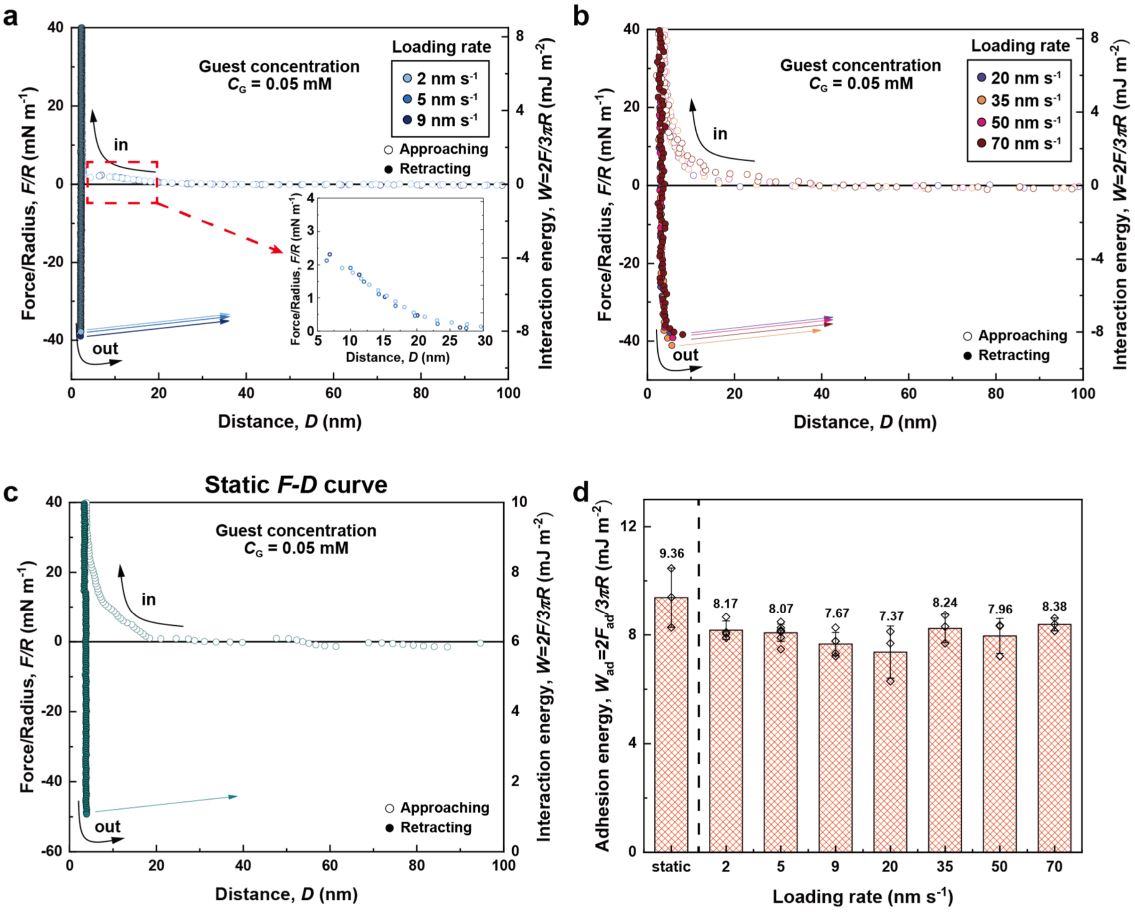

Supplementary Fig. 8 Effect of loading rate on the interaction forces. a Force-distance profile of symmetric β-CD-modified surfaces with 0.05 mM of DAd at different loading rate (2, 5 and 9 nm s^{-1}) and **b** further accelerated loading rate (20, 35, 50, and 70 nm s^{-1}). **c** Static force-distance profile measured by piezo system, which shows data points recorded after sufficient equilibration. **d** Adhesion energy as a function of loading rate (the error bars represent the s.e.m., where $n \geq 3$). Source data are provided as a Source Data file.

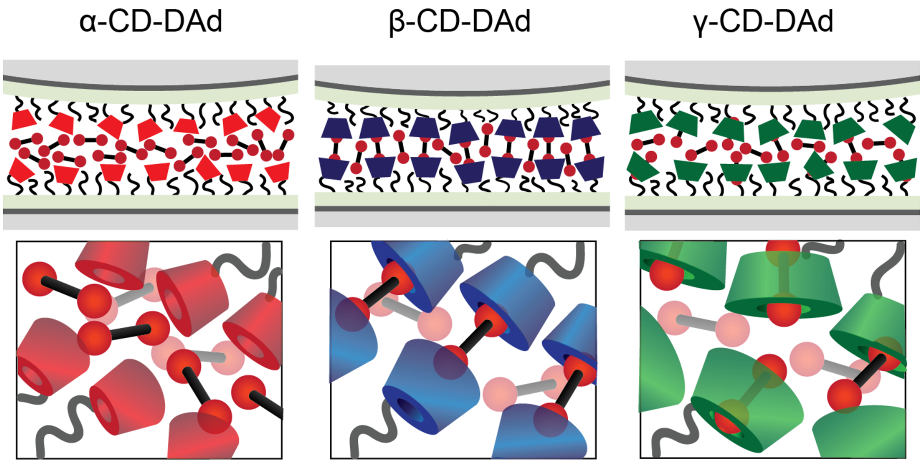

Supplementary Fig. 9 Host–guest interactions between α-, β-, and γ-CD-modified surfaces and DAd. The bridging interactions were maximized in the β-CD-DAd system owing to the compatibility of the cavity size of Ad.

Supplementary Notes

Supplementary Note 1: Possibility of intra-surface CD-DAd-CD bridging

We assumed that all ditopic adamantane molecules (DAd) fully formed 1:1 inclusion complex with cyclodextrins on surfaces in QCM-D measurement. Thus, we could calculate the surface density of 0.21 nm^{-2} and the distance of between two adjacent CDs (= 2.36 nm). We designed the DAd has stiff and short bipyridinium backbone, which has fully extended chain length of 2.05 nm (Supplementary Fig. 1a).

This chain length of the GPTMS linker was considered sufficiently short to prevent the DAd from forming inclusion complexes with CDs at same surface (Supplementary Fig. 1b). Although the bending of GPTMS linker is possible, geometrically, high bending angle (~44°) is required for intra-surface bridging to occur. The bending energy (E_{bend}, kcal mol^{-1}) given by:[3,4]

$$E_{\text{bend}} = 0.043838(k_\theta/2)(\theta - \theta_0)^2$$

where 0.043838 is the conversion factor, k_θ is the bending force constant, θ is the instantaneous bond angle (°), and θ_0 the initial equilibrium angle (°). In Supplementary Fig. 1c, the k_θ of C–C–O (**1, 2, 4**), C–O–C (**3**), C–C–C (**5**), and C–C–Si (**6**) is 0.70, 0.77, 0.45, and 0.40 mdyn Å rad^{-2}, respectively. We could calculate the minimum value of E_{bend} ~4.12 kcal mol^{-1} or ~7 k_BT at RT (from $\Delta\theta_1$, $\Delta\theta_2$, $\Delta\theta_4$ = 6.1°, $\Delta\theta_3$ = 5.5°, $\Delta\theta_5$ = 9.5°, and $\Delta\theta_6$ = 10.7°).

Therefore, the binding of both end groups of DAd to two adjacent CDs at same surface will be geometrically and energetically unfavorable due to the high energy barrier of bending.

Supplementary Note 2: Equilibrium of host-guest inclusion complex

Formation of Host–Guest complex can be expressed as following reaction formula:

$$[\mathrm{H}] + [\mathrm{G}] \leftrightharpoons [\mathrm{H:G}]$$

where [H], [G] and [H:G] are the concentration of cyclodextrin as host at the surface, ditopic adamantane molecule as guest in the bulk solution, and the host–guest inclusion complex at the surface, respectively.

We derived the binding constant of our systems as follows:

Rate of adsorption (r_a):

$$r_a = k_a[\mathrm{H}][\mathrm{G}] = k_a([\mathrm{H}]_0 - [\mathrm{H:G}])[\mathrm{G}]$$

where k_a is the adsorption rate constant and $[\mathrm{H}]_0$ is an initial concentration of host molecules on surface,

Rate of desorption (r_d):

$$r_d = k_d[\mathrm{H:G}]$$

where k_d is the desorption rate constant,

In equilibrium,

$$k_a([\mathrm{H}]_0 - [\mathrm{H:G}])[\mathrm{G}] = k_d[\mathrm{H:G}]$$

The binding constant of the complex (K) is given by:

$$K = \frac{k_a}{k_d} = \frac{[\mathrm{H:G}]}{([\mathrm{H}]_0 - [\mathrm{H:G}])[\mathrm{G}]}$$

The equation is simplified by:

$$\frac{[\mathrm{H:G}]}{[\mathrm{H}]_0} = \frac{K[\mathrm{G}]}{1 + K[\mathrm{G}]}$$

The ratio of $[\mathrm{H:G}]/[\mathrm{H}]_0$ can be substituted with the ratio of adhesion energy ($W_{\mathrm{ad}}/W_{\mathrm{ad,max}}$) as function of [G]. The $W_{\mathrm{ad,max}}$ can be determined by the adhesion energy at fully formed 1:1 inclusion complex with cyclodextrins on surfaces. Consequently, the relation between the adhesion energy and the concentration of guest molecule is given by:

$$W_{ad} = \frac{W_{ad,max}K[\mathrm{G}]}{1 + K[\mathrm{G}]} \qquad \text{Supplementary Equation (1)}$$

The K of $2.66 \times 10^5\ \mathrm{M}^{-1}$ ($\Delta G \sim 12.5\ k_{\mathrm{B}}T$) was determined by the fitting (Supplementary Fig. 5).

Supplementary References

1. Ryzhakov, A. *et al.* Self-Assembly of Cyclodextrins and Their Complexes in Aqueous Solutions. *J. Pharm. Sci.* **105**, 2556-2569 (2016).
2. Tsukruk, V. V., Luzinov, I. & Julthongpiput, D. Sticky Molecular Surfaces: Epoxysilane Self-Assembled Monolayers. *Langmuir* **15**, 3029-3032 (1999)
3. Allinger, N. L. Conformational analysis. 130. MM2. A hydrocarbon force field utilizing V1 and V2 torsional terms. *J. Am. Chem. Soc.* **99**, 8127-8134 (1977).
4. U. Burkert and N. L. Allinger "*Molecular Mechanics*", American Chemical Society, Washington, D.C., MD 1982.
